# The Immune Regulatory Role of Protein Kinase CK2 and Its Implications for Treatment of Cancer

**DOI:** 10.3390/biomedicines9121932

**Published:** 2021-12-17

**Authors:** Huixian Hong, Etty N. Benveniste

**Affiliations:** Department of Cell, Developmental and Integrative Biology, University of Alabama at Birmingham, Birmingham, AL 35294, USA; huixian@uab.edu

**Keywords:** protein kinase CK2, innate immunity, adaptive immunity, signaling pathways, CK2 inhibitors, cancer therapy

## Abstract

Protein Kinase CK2, a constitutively active serine/threonine kinase, fulfills its functions via phosphorylating hundreds of proteins in nearly all cells. It regulates a variety of cellular signaling pathways and contributes to cell survival, proliferation and inflammation. CK2 has been implicated in the pathogenesis of hematologic and solid cancers. Recent data have documented that CK2 has unique functions in both innate and adaptive immune cells. In this article, we review aspects of CK2 biology, functions of the major innate and adaptive immune cells, and how CK2 regulates the function of immune cells. Finally, we provide perspectives on how CK2 effects in immune cells, particularly T-cells, may impact the treatment of cancers via targeting CK2.

## 1. Introduction

The notion that CK2 is implicated in the pathogenesis of a plethora of diseases is well-established [1]. First discovered by George Burnett and Eugene P. Kennedy more than 6 decades ago [2], the field of CK2 biology has exploded with studies investigating phosphorylation targets of CK2, the cellular biology of CK2, and the important roles of CK2 in normal biological processes and disease pathogenesis. The importance of CK2 in tumorigenesis has been revealed in solid and hematologic tumors [3]. CK2 is consistently highly expressed in cancer cells and its anti-apoptotic, pro-migration and pro-proliferation functions contribute to the development of various types of cancers [4]. Recent studies on the involvement of CK2 in cells of the immune system indicates that CK2 plays a critical role not only in normal physiological functions of innate and adaptive immune cells, but also immune cells in aberrant pathological conditions [5]. There is increasing interest in using CK2 inhibitors for the treatment of various cancers. In this regard, an appreciation of how CK2 regulates the function of immune cells that are important for mounting immune responses against cancers needs to be considered to understand the implications of treatment strategies such as CK2 inhibition.

## 2. Protein Kinase CK2

### 2.1. CK2 Structure and Function

CK2 is usually a tetrameric kinase consisting of two catalytic (CK2α and/or CK2α′) and two regulatory (CK2β) subunits [6]. The catalytic subunits, CK2α and CK2α′, are encoded by *CSNK2A1* and *CSNK2A2* genes, respectively, while the regulatory subunit is encoded by the *CSNK2B* gene. CK2α and CK2α′ share 90% similarity within their catalytic domains [7]. There is only one form of CK2β characterized thus far, which is quite conserved among species [8,9,10]. The structural backbone of the tetrameric CK2 holoenzyme is a combination of two catalytic subunits (αα′, α′α′ or αα) and a dimer of two regulatory β subunits [6]. The β subunit is unstable and can dissociate from the tetrameric holoenzyme under physiological conditions [6]. CK2β plays a role in maintaining the stability of the tetramer, specificity towards target substrates and the ability of the CK2 tetramer to transit through cell membranes [11,12]. 

CK2 is constitutively active and can phosphorylate either serine/threonine or tyrosine residues, and the number of its target substrates are more than five hundred [1,11]. The phosphorylation function of CK2 occurs via recognizing its consensus sequence (S-X-X-E/D/Yp/Sp) [13,14]. CK2 is essential for embryonic development as mice with deletion of CK2α or CK2β are not viable [15,16,17]. CK2 is involved in a variety of physiological and pathological cellular processes and signaling pathways. These include transcription, translation, cell cycle progression, cell growth, cell survival, cell proliferation, cell morphology changes, protein stability and degradation, tumor progression and suppression and angiogenesis [18,19]. In addition, CK2 is implicated in the pathogenesis of neoplastic disorders, inflammatory diseases, autoimmune and infectious disorders [1,5].

### 2.2. Signaling Pathways Regulated by CK2

CK2 regulates a number of signaling pathways involved in immune cell development and function, including (1) the Phosphatidylinositol 3-Kinase (PI3K)/AKT/Mammalian Target of Rapamycin (mTOR) pathway; (2) the Nuclear Factor-κB (NF-κB) signaling pathway; and (3) the Janus Kinase (JAK)/Signal Transducers and Activators of Transcription (STAT) pathway [1].

**Figure 1:**
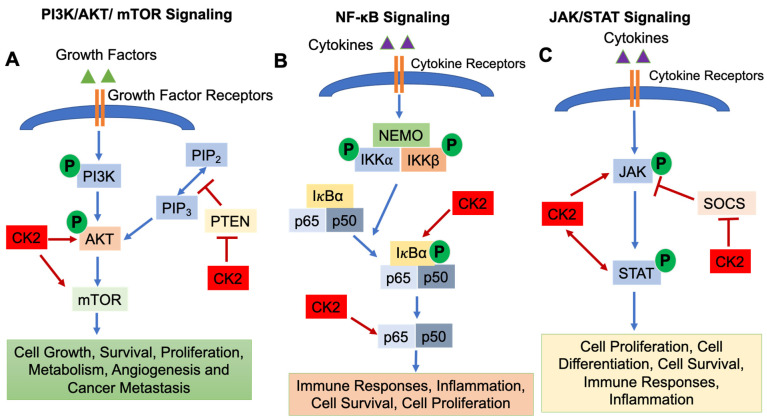
Signaling Pathways Regulated by CK2. The PI3K/AKT/mTOR (**A**), NF-κB (**B**) and JAK/STAT (**C**) signaling pathways are shown. CK2 involvement is shown as arrows (enhanced function) or bars (suppressive function). P: Phosphorylation.

The PI3K/AKT/mTOR pathway is essential for cell growth, survival, proliferation, metabolism, angiogenesis and cancer metastasis [20,21]. The PI3K/AKT/mTOR pathway is activated by binding of growth factors to receptors on the cell surface, which results in the phosphorylation of PI3K and activation of downstream molecules AKT and mTOR. mTOR then translocates into the nucleus and regulates target gene transcription [22]. Phosphatase and Tensin Homolog deleted on Chromosome 10 (PTEN) functions as a negative regulator to inhibit PI3K/AKT/mTOR signaling [23] (Figure 1A). CK2 is involved in activating the PI3K/AKT/mTOR pathway in several ways. CK2α and CK2β subunits physically interact with AKT, which results in enhanced activity of AKT [24]. Di Maira et al., demonstrated that CK2 can directly phosphorylate AKT at Ser129 which results in AKT activation [25,26]. Another critical role of CK2 in modulating the PI3K/AKT/mTOR signaling pathway is via regulating the activity of PTEN [11]. CK2 phosphorylates Ser370, Thr366 and Ser385 of the PTEN carboxy-terninus, which causes degradation of PTEN and suppresses PTEN phosphatase activity [27,28,29], thus promoting activation of PI3K/AKT/mTOR signaling. In addition, mTOR is a direct target of CK2 in human glioblastoma cells, and inhibition of CK2 by siRNA results in subsequent autophagic cell death [30]. Zheng et al., demonstrated that inhibition of CK2 using the inhibitor CX-4945 suppressed AKT activation and diminished adhesion and migration of human glioblastoma cells [31]. 

The NF-κB signaling pathway is activated by variety of stimuli, i.e., cytokines and growth factors binding their cell surface receptors [32]. The IκB kinase (IKK) complex triggers the phosphorylation and subsequent degradation of IκBα, resulting in the translocation of NF-κB transcription factors (p65 and p50) into the nucleus and subsequent gene transcription [32]. The NF-κB signaling pathway is indispensable for inflammatory responses and plays a critical role in the development and progression of cancers, both solid cancers and hematologic malignancies [33]. In addition, NF-κB signaling is important for the survival, activation, and differentiation of innate and adaptive immune cells, and deregulation of the NF-κB pathway is implicated in the pathogenesis of various inflammatory diseases [32]. CK2α promotes the phosphorylation of IκBα, an NF-κB inhibitor [1,34], which causes the degradation of IκBα, resulting in the release of p65 and/or p50 and translocation into the nucleus [1]. Moreover, CK2 directly phosphorylates the NF-κB p65 subunit on Ser529, which enhances its transcriptional activity [1,35] (Figure 1B). 

The JAK/STAT signaling pathway is essential for incorporating signals from cell surface receptors to the nucleus [36]. JAKs are tyrosine kinases which bind to the cytoplasmic domains of many cytokine/growth factor receptors [36]. When cytokines and growth factors bind to and activate their receptors, JAKs phosphorylate each other and phosphorylate tyrosine residues in the cytoplasmatic domain of the receptor. STAT transcription factors are then recruited to the receptor complex, phosphorylated by JAKs, form dimers, translocate into the nucleus and initiate gene transcription [37,38]. The initiation, duration and termination of the JAK/STAT signaling pathway are regulated by Suppressors of Cytokine Signaling (SOCS) proteins, which function in a negative feedback loop to suppress the JAK/STAT pathway [36] (Figure 1C). JAK/STAT signaling is involved in various cellular events, i.e., development of the immune system [39], cell survival, cell proliferation and angiogenesis [1,40]. CK2 has been shown to be involved in regulating the JAK/STAT signaling pathway. Inhibition of CK2 using CX-4945 resulted in decreased phospho-STAT3 levels in multiple myeloma and mantle cell lymphoma cells as well as suppression of STAT3 target genes (*Cyclin D1, IL-6*) [41]. Zheng et al., demonstrated that inhibition of CK2 by small interfering RNAs or the CK2 inhibitor TBB inhibited STAT3, STAT1, STAT5, and JAK2 phosphorylation/activation as well as expression of the *SOCS3* gene [42]. Furthermore, JAK2 is a substrate for CK2 as CK2 directly phosphorylates human JAK2 at sites T308, T310, and T817 [42]. Another study demonstrated that CK2 is essential for the activation of STAT3 which contributes to the survival of glioblastoma cells [31]. Taken together, CK2 is a critical activator of the JAK/STAT signaling pathway. 

### 2.3. CK2 Inhibitors

Currently available CK2 inhibitors can be divided into ATP-competitive CK2 inhibitors, allosteric compounds and bi-functional inhibitors based on their mechanisms of action [1]. 

The development of ATP-competitive CK2 inhibitors is based on the feature that the ATP pocket of CK2 is very small and narrow which makes it easy to make a stable interaction with small compounds [43,44]. The most representative of ATP-competitive CK2 inhibitors are CX-4945 [(5-(3-chlorophenyl)amino)-benzo(c)-2,6-naphthyridine-8-carboxylic acid)] and CIGB-300, which are in clinical trials for cancer therapy [1]. ATP-competitive CK2 inhibitors also include TBB (4,5,6,7-tetrabromo-1H- benzotriazole), DMAT (2-dimethylamino-4,5,6,7-tetrabromo- 1H-benzimidazole), Apigenin (4′,5,7,-trihydroxyflavone) and Emodin (1,3,8-trihydroxy-6-methyl-antraquinone), although these have some off-target effects. Table 1 lists commonly used ATP-competitive CK2 inhibitors and their clinical trial status, if applicable. 

Allosteric compounds, i.e., BPN14770 [45], act at the interface between α and β subunits and disrupt their normal functions, however, the exact mechanism of this inhibitor is still unclear [1,46,47]. Bi-functional inhibitors suppress CK2 by competing for both the phosphate donor nucleotide and the phosphate acceptor peptide [1,48,49]. 

**Table 1:** Commonly Used ATP-competitive CK2 Inhibitors.

Compound	Clinical Trials	References
CX-4945	Phase I/II for basal cell carcinoma and medulloblastoma	[1,44]
CIGB-300	Phase I/II for cervical cancer	[1,44,50]
TBB	N/A	[44]
DMAT	N/A	[44]
Apigenin	N/A	[44]
Emodin	N/A	[44]

N/A: Not Applicable.

## 3. Role of the Immune System in Health and Disease

**Figure 2:**
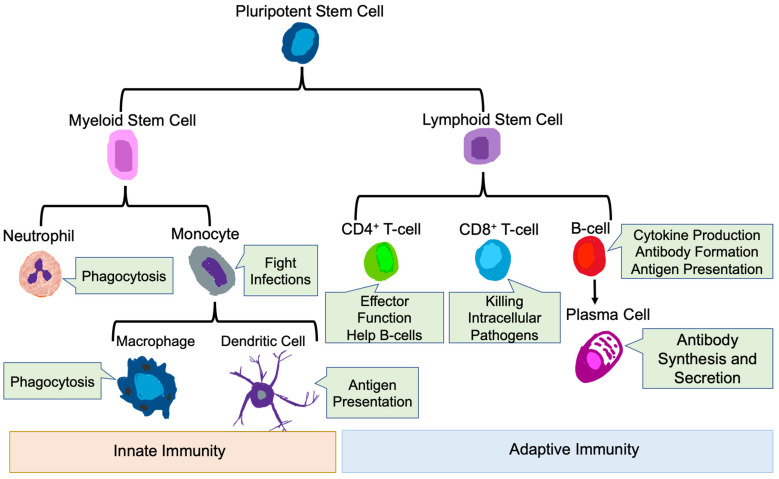
Innate and Adaptive Immune Cells. Development of innate and adaptive immune cells as well as their key functions are shown.

The mammalian immune system protects the host from pathogenic microbes via effector mechanisms to eliminate invading microbes, while at the same time avoiding the destruction of self-tissues [51]. The immune system is broadly classified into innate and adaptive immune systems [52]. The main immune cells of the innate immune system are neutrophils, macrophages, monocytes, and dendritic cells (DC) [53,54], while the main immune cell types in the adaptive immune system are T-cells and B-cells [51] (Figure 2).

### 3.1. Innate Immune Cells

#### 3.1.1. Neutrophils

As primary responders during infection and inflammation [55,56], neutrophils are the most abundant of circulating innate immune cells and are essential for defense against invading microorganisms [57]. Neutrophils execute their anti-infectious functions via the activation of specific receptors, migration into tissues, pathogen sensing, and ultimate killing of pathogens [56]. In addition, neutrophils phagocytose bacteria and other microorganisms and subsequently kill them via the production of reactive oxygen species [58]. Neutrophils also regulate the function of other immune cells by the production of pro- inflammatory cytokines and chemokines such as IL-1b, TNF-a, IL-6, CXCL2 and CCL2 [59]. Thus, neutrophils play an essential role in regulating inflammatory and immune responses.

#### 3.1.2. Monocytes and Macrophages

Monocytes are a type of mononuclear phagocyte that mainly resides in the circulation [60]. Monocytes develop in the bone marrow from hematological precursors [61]. Circulating monocytes have many functions, including pro-inflammatory, anti-inflammatory, antigen presentation and tissue remodeling and are essential innate cells in pathological conditions, i.e., inflammation [61]. Macrophages are generated and differentiated from circulating monocytes upon entry into inflamed tissues [62]. Macrophages have both detrimental and protective functions that are ascribed to polarization to different phenotypes based on the cytokine microenvironment, broadly termed pro-inflammatory (M1) and anti-inflammatory (M2) [63]. In vitro, macrophages can be polarized to the pro-inflammatory phenotype by IFN-γ and GM-CSF, express high levels of MHC Class II, IL-12, IL-6, IL-1β, TNF-α IL-23, CXCL10, CCL2 and iNOS, and participate in the induction of pathogenic responses [64,65,66]. Stimulation with IL-4, IL-13 and M-CSF in vitro induces anti-inflammatory macrophages, which express mannose receptors (CD206), Arginase 1, and high levels of IL-10 and TGF-β [67,68]. Pro-inflammatory macrophages have pathogen killing functions, promote tissue injury and have anti-tumorigenic properties, while anti-inflammatory macrophages promote clearance of apoptotic cells, are involved in wound healing, and promote tumor progression and metastasis [67,69,70]. These anti-inflammatory macrophages in the tumor setting are referred to as tumor associated macrophages (TAMs) [70].

#### 3.1.3. Dendritic Cells

Dendritic cells (DCs) are a subset of monocyte-derived immune cells with diverse plasticity, which differentiate from circulating monocytes once they enter tissues [71]. DCs are professional antigen presenting cells and play an essential part in initiating and regulating innate and adaptive immune responses [71]. Their primary function is to integrate biological signals from injured tissues and capture, process, and present antigen from injured sites to naïve T-cells within secondary lymphoid organs [72,73]. A new classification system divides DCs into conventional DCs, plasmacytoid DCs, monocyte-derived DCs (MoDCs) and Langerhans cells, based on the ontogeny of the cells [72]. 

### 3.2. Adaptive Immune Cells

#### 3.2.1. T-Cells

**Figure 3:**
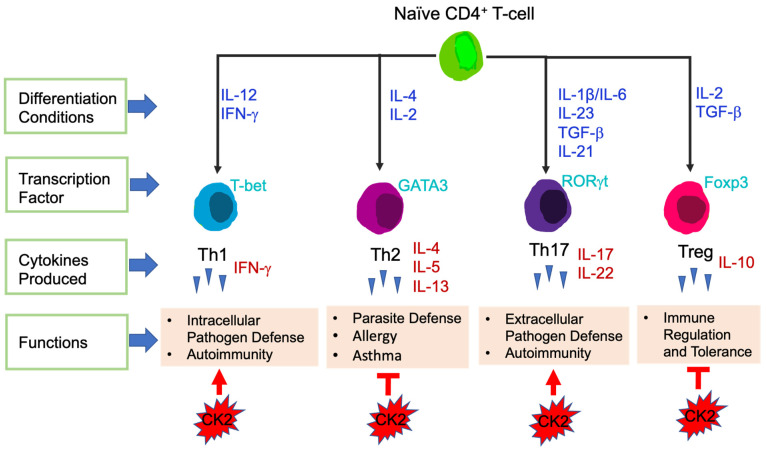
Differentiation of CD4^+^ T-cell Subsets. Differentiation conditions by cytokines, key transcription factors expressed and main functions are shown. CK2 involvement in CD4^+^ T-cell subsets is shown at the bottom. Arrows represent enhanced function and bars represent suppressive function.

T-cells are the central effector cells in adaptive immune responses, including CD4^+^ T-cells and CD8^+^ T-cells [74]. CD4^+^ T-cells, also called CD4^+^ helper T-cells, recognize exogenous antigen via MHC class II molecules [75]. Based on the transcription factors and cytokines expressed, CD4^+^ T-cells are divided into different subsets: T helper 1 (Th1), Th2, Th17 and T Regulatory (Tregs) (Figure 3). The key transcription factors and signature cytokines of each CD4^+^ T-cell subset are as follows: Th1: T-bet, IFN-γ; Th2: GATA3, IL-4, IL-5, IL-13; Th17: RORγt, IL-17, IL-22; and Treg: Foxp3, IL-10 [76,77]. Th1 cells are essential for host defense against intracellular pathogens and contribute to the pathogenesis of autoimmune diseases [78]. Th2 cells function to fight against parasite infections and also are critical in the development of allergy and asthma. Th17 cells are involved in autoimmunity as well as host defense against extracellular bacteria and fungi [76]. Tregs maintain immune tolerance, have immunosuppressive functions, and in the context of cancer, Tregs suppress anti-tumor immune responses [77].

#### 3.2.2. B-Cells

**Figure 4:**
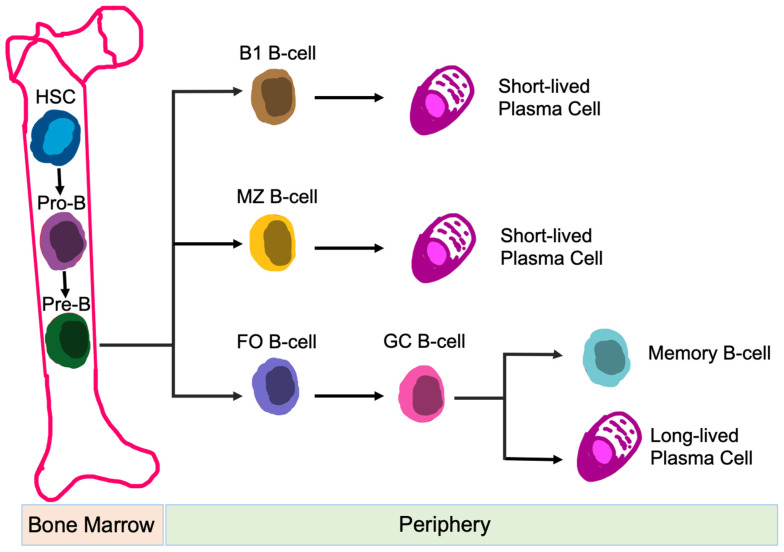
Development of B-cell Subsets. Differentiation of different B-cell subsets is shown. Abbreviations: HSC, hematopoietic stem cell; MZ, marginal zone; FO, follicular; and GC, germinal center.

B-cells are responsible for humoral immune responses [79]. In addition to antibody formation, B-cell functions also include: antigen presentation [80], regulation of T-cell differentiation and survival [81], and production of both pro-inflammatory and anti-inflammatory cytokines [82]. B-cells are generated from hematopoietic precursor cells within the bone marrow. Once development is complete, B-cells circulate into the periphery and enter secondary lymphoid organs, i.e., spleen, lymph nodes, tonsils, Peyer’s patches, and mucosal tissues [83]. Naïve B-cells are categorized into B-1 B-cells, follicular (FO) B-cells, and marginal zone (MZ) B-cells, based on their location, migration potential, and activation fashion [84] (Figure 4). Dysfunction of B-cells contributes to autoimmunity and immunological disorders [85]. 

## 4. CK2 Regulates Immune Cell Function

### 4.1. Innate Immunity

#### 4.1.1. CK2 Function in Neutrophils, Monocytes and Macrophages

The role of CK2 in regulating the function of innate immune cells is not well understood. To address this, the Lenz group generated a conditional deletion model (*Csnk2 floxed Lyz2*-Cre mice), in which the catalytic CK2α subunit was deleted in myeloid cells (neutrophils, monocytes and macrophages) [86]. First, this study revealed that CK2α does not affect the development of myeloid cells, as determined by evaluating the numbers of F4/80^+^ macrophages, Ly6C^+^ monocytes and neutrophils. Next, they investigated the impact of CK2α deficiency with respect to response to pathogens, utilizing systemic *Listeria monocytogenes* infection. It was demonstrated that the recruitment and anti-bacterial activity of myeloid cells was enhanced in the absence of CK2α, resulting in reduced bacterial burden. This was associated with an increased accumulation of neutrophils, macrophages and monocytes early after *Listeria monocytogenes* infection. The impact of CK2α on the activation and bactericidal potential of myeloid cells was then assessed. Class II MHC expression was enhanced in the absence of CK2α, as was phagosome maturation. These two functional changes may account for the improved bactericidal function of myeloid cells lacking CK2α. The authors were not able to precisely elucidate how CK2α influences these functions, other than to suggest that CK2α blunts early myeloid cell activation and accumulation at sites of infection. This study demonstrated that expression of CK2α in myeloid cells was detrimental to the host in the setting of systemic *Listeria monocytogenes* infection, suggesting that targeting CK2 may be beneficial in the context of pathogenic infections [86]. 

The study described above is the only one to examine the role of CK2α in myeloid cells by conditional deletion in vivo. Other studies have examined various functions of CK2 utilizing in vitro experiments. IL-1β is a potent pro-inflammatory cytokine implicated in many pathological conditions [87]. Cells of the macrophage/monocyte lineage are the major sources of IL-1β in vivo. The Nikolajczyk group demonstrated that CK2 is critically involved in transcription of the *IL-1β* gene in monocytes in response to a variety of stimuli [88,89]. The IL-1β promoter is packaged into a non-transcribed “poised” configuration in the absence of any stimulation, which includes the association of two transcription factors, PU.1 and C/EBPβ. Upon LPS stimulation, a third transcription factor, IRF-4, is recruited to the promoter, allowing for transcription of the IL-1β promoter and subsequent IL-1β expression [88]. Importantly, the phosphorylation of PU.1 at serine 148 by CK2 is indispensable for the recruitment of IRF-4 to the IL-1β promoter in monocytes, and CK2 itself associates with the IL-1β promoter upon cell stimulation [88]. Inclusion of CK2 inhibitors such as apigenin or emodin abrogated IL-1β induction, demonstrating a critical role for CK2 in driving IL-1β expression in monocytes. In another study, Jung et al., demonstrated that diosgenin, a precursor of steroid hormones, inhibited CK2α expression in the nucleus of LPS/IFN-γ treated murine peritoneal macrophages and inhibited LPS/IFN-γ induction of a number of pro-inflammatory mediators including iNOS, ROS, IL-1 and IL-6 [90]. Diosgenin inhibited CK2 activation as well as JNK, NF-κB and AP-1 activation, and the authors speculate that inhibition of these kinases and transcription factors contributed to decreased production of pro-inflammatory mediators by macrophages [90]. Collectively, these two in vitro studies indicate that CK2 activation in monocytes/macrophages in response to cell stimulation leads to the induction of pro-inflammatory responses.

A study by Hashimoto et al., examined the impact of CK2 on modulating myeloid cells in the tumor microenvironment [91]. Utilizing BMS-211, a prodrug of the parent pan-CK2 inhibitor BMS-699, they demonstrated potent anti-tumor effects in combination with immunotherapy in a number of pre-clinical tumors models. The beneficial effect of CK2 inhibition was not the result of direct tumor toxicity, rather, due to effects on myeloid cells, especially TAMs and polymorphonuclear myeloid-derived suppressor cells (PMN-MDSC). CK2 inhibition resulted in a decrease of TAMs and PMN-MDSC with tumor-promoting properties in the tumor microenvironment, in part by interfering with myeloid cell differentiation. This study concludes that targeting CK2 may be a valuable addition to immune-based therapeutics in the treatment of cancer. 

#### 4.1.2. CK2 Function in Dendritic Cells (DCs)

Findings from de Bourayne et al., highlight the role of CK2 in regulating human MoDCs in response to chemicals involved in the pathogenesis of allergic contact dermatitis [92]. This study demonstrated that CK2 kinase activity is essential for the maturation of MoDCs in response to allergic contact dermatitis related chemicals as assessed by the expression of surface markers including CD86, CD83, CD40, PD-L1 and MHC Class II. This response was abrogated by the inclusion of the CK2 inhibitor CX-4945. In addition, CK2 drives the production of numerous pro-inflammatory cytokines/chemokines by MoDCs in response to allergic contact dermatitis related chemicals, including IL-1β, IL-6, TNF-α, CCL4 and CXCL8. Co-incubation of DCs with CD4^+^ T-cells induces their activation and polarization to the Th1 phenotype, which is also inhibited by the inclusion of CX-4945 [92]. Reverendo et al., recently elucidated a critical role of CK2 in regulating the immune-stimulatory functions of DCs [93]. They demonstrated that CK2 phosphorylation of MAF1 homolog negative regulator of Poll III (MAF1), and its subsequent exit from the nucleus, contributed to a strong elevation of RNA polymerase III, which then promoted the translation of DC mRNAs coding for a number of co-stimulatory molecules and pro-inflammatory cytokines. These activities are essential for DC functionality, especially as related to naïve T-cell priming [93]. These two studies highlight the importance of CK2 in DC functionality by regulating aspect of important inflammatory processes.

### 4.2. Adaptive Immunity

#### 4.2.1. CK2 Function in CD4^+^ T-Cells

The most detailed information on CK2 in the context of the immune system comes from work examining its influence on CD4^+^ T-cells. CD5 is a transmembrane receptor that regulates a number of T-cell functions [94]. There is a signaling domain within the cytoplasmic tail of CD5 which is a CK2 binding/activation domain (CD5-CK2BD). The Raman group demonstrated that disruption of CD5-CK2 signaling diminished the development of experimental autoimmune encephalomyelitis (EAE), a pre-clinical model of Multiple Sclerosis, which is T-cell dependent [95]. This was due to a decreased frequency of pathogenic CD4^+^ T-cells expressing both IFN-γ and IL-17 [95]. Subsequently, the Raman group generated a knock-in mouse that expressed a CD5 protein containing a microdeletion with selective inability to interact with CK2 [96]. Intact CD5-CK2 signaling was critical for the development of CD4^+^ Th2 and Th17 cells, but not CD4^+^ Th1 cells, by regulating the threshold for T-cell responsiveness [96]. These studies demonstrated the essential role of the CD5-CK2 pathway in regulating the development and differentiation of CD4^+^ T-cells. 

The direct involvement of CK2 in CD4^+^ T-cells was first demonstrated by Ulges el. al., in 2015. They generated a murine model in which the regulatory subunit CK2β was specifically deleted in CD4^+^ Treg cells [97]. This study revealed that CK2β was required for a specific population of Tregs to suppress CD4^+^ Th2-cell mediated allergic immune responses in the lung [97]. In subsequent studies, Ulges et al., as well as the Benveniste group explored the effect of CK2 in regulating the differentiation of naïve CD4^+^ T-cells into different functional subsets. First, the levels of CK2 subunits and kinase activity were examined. Naïve CD4^+^ T-cells constitutively express very low levels of CK2α, CK2α′ and CK2β, however, expression was induced upon T-cell stimulation [98]. Furthermore, CK2 kinase activity was also induced upon T-cell stimulation [98]. As mentioned previously, Th17 cells in general are pro-inflammatory, while Tregs exhibit immunosuppressive functions [77]. Both groups found that inhibition of CK2 kinase activity in CD4^+^ T-cells using CX-4945 promoted the differentiation of Treg cells at the expense of Th17 cells [98,99], in part by suppression of PI3K/Akt/mTOR activation and STAT3 phosphorylation [98]. These findings indicate that CK2 promotes Th17 cell differentiation while suppressing the generation of Treg cells, thus skewing CD4^+^ T-cells to have pro-inflammatory functions. In addition, both groups found that systemic inhibition of CK2 using CX-4945 ameliorated the severity of EAE due to the suppression of pathogenic Th17 cells and expansion of Treg cells [98,99]; Figure 3. 

To elucidate the role of CK2 in the EAE model, genetic approaches were utilized. Ulges et al., demonstrated that deletion of CK2β specifically in CD4^+^ T-cells resulted in significant protection in the EAE model, which was associated with suppression of Th17 cells and promotion of Treg cells in vivo [99]. Gibson et al., generated a mouse model in which CK2α was specifically deleted in CD4^+^ T-cells [98,100]. The severity of EAE was significantly decreased in mice lacking CK2α in CD4^+^ T-cells, and this was associated with a significant defect in Th17 cell polarization and a reciprocal increase in Tregs in vivo [98,100]. These in vivo genetic models indicate that both subunits of CK2, CK2α and CK2β, are involved in promoting Th17 cell differentiation and suppressing Tregs, leading to autoimmune neuroinflammation; Figure 3.

To further explore the influence of CK2α in a different inflammatory disease setting, Yang et al., utilized a T-cell induced colitis model, which serves as a pre-clinical model for Crohn’s Disease (CD) [101]. Results from this study demonstrated that the absence of CK2α in CD4+ T-cells resulted in significantly less colitis disease severity and intestinal inflammation, which was associated with decreased Th17 cells producing IL-17A, Th1 cells producing IFN-γ and double positive IL-17A, IFN-γ producing CD4^+^ T-cells in the spleen and colon. CK2α was shown to be essential for the phosphorylation of STAT4 in CD4^+^ T-cells upon T-cell activation and control expression of the IL-12 receptor, thus regulating IL-12/STAT4 pathway activation and promoting Th1 cell differentiation [101]. In addition, CK2α was shown to be important in controlling CD4^+^ T-cell responses by regulating NFAT2, which is vital for T-cell activation and proliferation. These findings indicate that CK2α contributes to the pathogenesis of colitis by promoting CD4^+^ T-cell proliferation and Th1 and Th17 cell responses (Figure 3), and that targeting CK2 may be a novel therapeutic treatment for patients with CD.

#### 4.2.2. CK2 Function in B-Cells

The effect of CK2 on normal B-cell function has not been studied until recently, when Wei et al., generated mice lacking CK2α specifically in B-cells (using CD19-driven Cre recombinase) [102]. First, Wei et al., reported that CK2 subunit expression and kinase activity in naïve B-cells was very low, and inducible upon different stimulation conditions suggesting the involvement of CK2 in B-cell functions. In vivo, CK2α deficiency in B-cells resulted in an expansion of MZ B-cells and a reduction of transitional B-cells in murine spleen. CK2α promotes transitional B-cell development into MZ B-cells in spleen through regulating the magnitude of B-cell receptor (BCR) and Notch2 signaling. As such, in the absence of CK2α, reduced BCR signaling and elevated Notch2 signaling activation increased MZ B-cell differentiation. This study identifies the involvement of CK2α in normal B-cell development and differentiation, which may have implications for B-cell dysfunction in settings of cancer or inflammatory diseases.

## 5. Perspectives on the Use of CK2 Inhibitors for Cancer Therapy

It is well documented that CK2 expression is substantially enhanced in many solid tumors compared to normal tissues or cells, and this is also the case for hematologic tumors, including leukemia, lymphoma and multiple myeloma [103]. For the most part, expression of CK2 subunits is elevated, there is prominent nuclear localization of CK2 subunits, and CK2 kinase activity is enhanced [104,105,106,107,108]. This results in activation of oncogenic signaling pathways that are regulated by CK2, leading to many of the classical hallmarks of cancer, including cell proliferation, angiogenesis, invasion and metastasis, and resistance to cell death [109]. As such, there is much interest in targeting CK2 as a multi-therapeutic approach in both hematologic and solid tumors. Beneficial effects of various CK2 inhibitors have been observed in in vitro experiments, including suppressing proliferation and promoting apoptosis of transformed cells [110,111,112].

Another hallmark of cancer is the ability of cancer cells to evade the immune system [113]. Both innate and adaptive immune cells are found in the tumor microenvironment, and depending on their phenotype, can have anti-tumorigenic properties or suppress anti-tumor immune responses [114]. In this regard, a cautionary note on the use of CK2 inhibitors is raised given the role of CK2 on cells of the immune system. One example is the effect of CK2 inhibition in cells of the innate immune system. Inhibition of CK2 combined with immunotherapy in pre-clinical cancer models has a beneficial effect by affecting myeloid cells (PMN-MDSC and TAMs) in a manner that has an indirect anti-tumor effect [91]. In this case, CK2 inhibition would be viewed as favorable. However, inhibition of CK2 in DCs appears to suppress some of the immune-stimulatory functions of DCs that are anti-tumorigenic (proinflammatory cytokine production, T-cell polarization) [92,93]. Innate immune cells play important roles in the context of the tumor microenvironment with respect to inhibiting tumor growth [70,115], and CK2 has differential effects on these anti-tumorigenic properties that must be taken into consideration regarding CK2 inhibition. Another example is CK2 regulation of the CD4^+^ Th17/Treg axis. CK2α and CK2β promote the differentiation and function of Th17 cells at the expense of Tregs, which is associated with autoimmunity [98,100]. As such, CK2 inhibition in the autoimmune setting inhibits the differentiation of pathogenic Th17 cells and promotes the expansion of immunosuppressive Tregs, which is beneficial given the critical role of Tregs in self-tolerance [98,99,100]. However, Tregs suppress anti-tumor immune responses, promote tumor cell growth and enhance cell survival, and infiltration of large numbers of Tregs into tumor tissues is often associated with poor prognosis [116]. Systemic CK2 inhibition may have a beneficial effect on tumor cells themselves, but can promote the differentiation of Tregs, thereby having a deleterious influence in the setting of cancers. Another example of potential differential effects of CK2 on immune cells and cancer cells involves the Ikaros/CK2 axis. Ikaros, a transcription factor, represses expression of the *BCL2L1* gene, which encodes the anti-apoptotic protein Bcl-XL, in B-cell acute lymphoblastic leukemia (B-ALL). CK2, via phosphorylation of Ikaros, impairs this function of Ikaros, leading in part to resistance to doxorubicin treatment [117]. Inhibition of CK2 restores Ikaros-mediated repression of *BCL2L1* and enhances sensitivity of B-ALL cells to doxorubicin, demonstrating a beneficial effect of CK2 inhibition in this setting. Interestingly, a recent study demonstrated that in T-cells, Ikaros functions as a negative regulator of the cytokines GM-CSF and IFN-γ, thereby suppressing the development of pro-inflammatory CD4^+^ T-cells [118]. Given our findings of CK2 kinase activity in CD4^+^ T-cells [98,100], it will be of interest to study how CK2 affects the functionality of Ikaros in T-cells, how this may affect the polarization and function of these cells to have anti-or-pro-tumorigenic activity, and the consequences of CK2 inhibition. These studies provide more evidence on the complexity of the regulatory role of CK2 in normal immune cells and cancer therapy. In clinical trials utilizing CK2 inhibitors, it will be important to monitor the effect on immune cells in the tumor microenvironment to assess overall efficacy. Clearly, the biologic functions of CK2 are complex, and a deeper understanding of its function in immune and cancer cells will aid in establishing the effective use of CK2 inhibitors in solid and hematologic tumors. 

## Abbreviations

B-ALL	B-cell acute lymphoblastic leukemia
CX-4945	(5-(3-chlorophenyl)amino)-benzo(c)-2,6-naphthyridine-8-carboxylic acid)
CD	Crohn’s Disease
DMAT	2-dimethylamino-4,5,6,7-tetrabromo-1H-benzimidazole
DCs	Dendritic Cells
FO	Follicular
IL-1β	Interleukin-1β
IKK	IκB Kinase
JAK	Janus Kinase
MoDCs	Monocyte-derived DCs
MZ	Marginal Zone
mTOR	Mammalian Target of Rapamycin
NF-κB	Nuclear Factor-κB
PI3K	Phosphatidylinositol 3-Kinase
PTPs	Protein Tyrosine Phosphatases
PTEN	Phosphatase and Tensin Homolog deleted on Chromosome 10
STAT	Signal Transducers and Activators of Transcription
SOCS	Suppressors of Cytokine Signaling
TBB	4,5,6,7-tetrabromo-1H-benzotriazole
Th1	T helper 1
Th17	T helper 17
Tregs	T Regulatory Cells
TAMs	Tumor Associated Macrophages
